# Cdc14 phosphatase: warning, no delay allowed for chromosome segregation!

**DOI:** 10.1007/s00294-015-0502-1

**Published:** 2015-06-27

**Authors:** Félix Machín, Oliver Quevedo, Cristina Ramos-Pérez, Jonay García-Luis

**Affiliations:** Unidad de Investigación, Hospital Universitario Nuestra Señora de la Candelaria, Ctra del Rosario 145, 38010 Santa Cruz de Tenerife, Spain; Center for Chromosome Stability and Department of Biology, University of Copenhagen, 2200 Copenhagen, Denmark; Cell Cycle Group, MRC Clinical Sciences Centre, Imperial College London, Du Cane Road, London, W12 0NN UK

**Keywords:** Cdc14, *Saccharomyces cerevisiae*, rDNA, Anaphase bridges, Gross chromosomal rearrangements, Aneuploidy

## Abstract

Cycling events in nature start and end to restart again and again. In the cell cycle, whose purpose is to become two where there was only one, cyclin-dependent kinases (CDKs) are the beginning and, therefore, phosphatases must play a role in the ending. Since CDKs are drivers of the cell cycle and cancer cells uncontrollably divide, much attention has been put into knocking down CDK activity. However, much less is known on the consequences of interfering with the phosphatases that put an end to the cell cycle. We have addressed in recent years the consequences of transiently inactivating the only master cell cycle phosphatase in the model yeast *Saccharomyces cerevisiae*, Cdc14. Transient inactivation is expected to better mimic the pharmacological action of drugs. Interestingly, we have found that yeast cells tolerate badly a relatively brief inactivation of Cdc14 when cells are already committed into anaphase, the first cell cycle stage where this phosphatase plays important roles. First, we noticed that the segregation of distal regions in the chromosome arm that carries the ribosomal DNA array was irreversibly impaired, leading to an anaphase bridge (AB). Next, we found that this AB could eventually be severed by cytokinesis and led to two different types of genetically compromised daughter cells. All these previous studies were done in haploid cells. We have now recently expanded this analysis to diploid cells and used the advantage of making hybrid diploids to study chromosome rearrangements and changes in the ploidy of the surviving progeny. We have found that the consequences for the genome integrity were far more dramatic than originally envisioned.

## Role of Cdc14 in the resolution and segregation of sister chromatids

Cdc14 is a cell cycle phosphatase essential for the mitosis-to-G_1_ transition in *Saccharomyces cerevisiae*, the end of the cell cycle (reviewed in Stegmeier and Amon 2004; De Wulf et al. 2009; Mocciaro and Schiebel 2010; Uhlmann et al. 2011; Wurzenberger and Gerlich 2011; Meitinger et al. 2012; Weiss 2012). This protein belongs to a superfamily of dual-specificity phosphatases highly conserved in all eukaryotes, although their roles seem to have diverged during evolution. Yeast Cdc14 preferentially dephosphorylates CDK targets, which makes it the main antagonist of CDK in the cell. Although there are a number of mechanisms that counteract CDK actions during the cell cycle, Cdc14 triggers the final wave of synergistic events that completely eliminates the activity of CDK and other mitotic kinases. This is achieved by activating an alternative to the Cdc20-regulated Anaphase Promoting Complex (APC^Cdc20^), the APC^Cdh1^, as well as expressing and activating the CDK inhibitor Sic1. Thus, Cdc14 actions at the end of mitosis, coupled to the overall CDK inactivation, changes the phosphorylation/dephosphorylation ratio of most CDK targets in favor of the latter. Cdc14 is first activated shortly after anaphase starts by the action of APC^Cdc20^. Before that, Cdc14 is kept fully inactive through its binding to the nucleolar protein Net1 (Cfi1). This first wave of Cdc14 activation occurs through the FEAR (Cdc Fourteen Early Anaphase Release) network and takes place while the CDK activity is still high, contributing to the beginning of its decline. Shortly afterwards, a second and more potent Cdc14 activation by the Mitotic Exit Network (MEN) greatly accelerates the loss of CDK and ensures cytokinesis and the exit from mitosis into a new G_1_ for the mother and daughter cells.

Cdc14 activation by FEAR is dispensable for sustaining the growth of a yeast population, although affects cell viability (D’Amours et al. 2004). We now know that this loss of viability relates to the occurrence of aberrant chromosome segregation in anaphase. Thus, conditional mutants for MEN (the prototypical example being the thermosensitive allele *cdc15*-*2*) block cells with high CDK activity right before cytokinesis. However, sister chromatid resolution and segregation appears complete, even for challenging regions (see below) (D’Amours et al. 2004; Torres-Rosell et al. 2004; Machín et al. 2005; Quevedo et al. 2012). Accordingly, positioning of the centrosome-like microtubule organizing centers known as Spindle Pole Bodies (SPB) appears correct, and nuclear spindle microtubules look healthy and elongated (D’Amours et al. 2004; Machín et al. 2005; Jin et al. 2008; Quevedo et al. 2012). Importantly, in MEN mutants, the first wave of Cdc14 activation by FEAR still takes place, although Cdc14 becomes inactive and relocates to the nucleolus quickly (Stegmeier et al. 2002). In contrast, double mutants for both FEAR and MEN, or prevention of any Cdc14 activity by using the *cdc14*-*1* (or *cdc14*-*3*) thermosensitive allele (ts), leads to an anaphase block with incomplete positioning of the SPBs in the mother-to-daughter axis, unhealthy bended and broken spindles, lagging DAPI-stained DNA masses at the bud neck, a gross failure to segregate the nucleolus, and unresolved sister chromatids near the telomeres of several chromosome arms (D’Amours et al. 2004; Sullivan et al. 2004; Torres-Rosell et al. 2004; Ross and Cohen-Fix 2004; Machín et al. 2005; Jin et al. 2008; Clemente-Blanco et al. 2009). The molecular basis for all these failures is not completely understood, however, it is now clear that Cdc14 dephosphorylates, and thus activates, important players in the dynamics and stability of the anaphase spindle such as Fin1, Ase1, Sli15, Cin8, etc., [(Roccuzzo et al. 2015) and references therein]. Cdc14 also down-regulates transcription and, directly or indirectly, targets the condensin complex to the DNA (D’Amours et al. 2004; Machín et al. 2006; Clemente-Blanco et al. 2009). Condensin is in turn a master player in two important steps needed for sister chromatid resolution and segregation in eukaryotes. On the one hand, condensin drives the axial compaction of chromosome arms to make them short enough to avoid lagging DNA at the cytokinetic plane. On the other hand, condensin promotes the decatenation activity of topoisomerase II (Top2), essential for sister chromatid resolution (reviewed in Hirano 2012).

## Without Cdc14 the right arm of chromosome XII forms an anaphase bridge: the causes

**Fig. 1:**
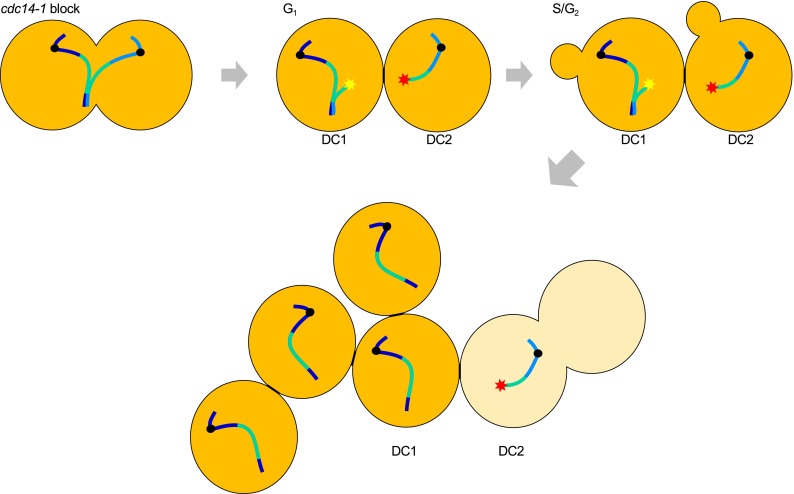
The structure and consequences of the chromosome XII right-arm anaphase bridge (cXIIr-AB) seen in *cdc14*-*1* haploid cells. Sister chromatids for chromosome XII are depicted in *light and dark blue*. Centromeres are *black circles* and the rDNA array is in *green* for both sisters. The cXIIr-AB comprises centromere-to-telomere partly unzipped sister chromatids with the rDNA being the source of non-resolution. The cXIIr-AB is present at the *cdc14*-*1* late anaphase block (*upper left dumbbell cell*). Release from the block leads to the breakage of the cXIIr-AB in a subpopulation of cells, leading to two daughter cells with distinct cXII content (*upper center dumbbell cell*). Daughter cell number one (DC1) will carry the acentric fragment of the broken sister chromatid together with the intact sister (*yellow star* the DSB is suitable to be repaired by BIR). Daughter cell number two (DC2) will carry the remaining centromere-containing broken sister (*red star* the DSB is not suitable to be repaired by BIR). DC2 will die shortly after the cXIIr-AB breakage since it lacks important genetic information present in the rDNA-to-telomere cXIIr region. Our data suggest that DC1 will survive genetically unchanged, ridding itself of the acentric fragment (*lower cells*)

An interesting finding in relation to the failure to resolve and segregate the sister chromatids in a *cdc14*-*ts* block is that not all chromosomes are impaired to the same extent. Thus, distal regions of the long chromosome arms, certain telomeres (but not all), and the ribosomal DNA array (rDNA) are particularly impeded. The rDNA has been studied in depth (Granot and Snyder 1991; D’Amours et al. 2004; Sullivan et al. 2004; Wang et al. 2004; Torres-Rosell et al. 2004; Machín et al. 2005; Torres-Rosell et al. 2005; Machín et al. 2006; Tomson et al. 2006; Geil et al. 2008; Clemente-Blanco et al. 2009). The rDNA is a highly repetitive region, comprising 100–200 head-to-tail copies of a ~9 kb unit, located on the right arm of the chromosome XII (Petes 1979). This arm (cXIIr hereafter) is relatively large even without taking into account the rDNA. Including the rDNA, cXIIr becomes by far the largest arm in the yeast genome. In theory, with the average degree of compaction for the yeast genome, the distance reached between the two SPBs in late anaphase is insufficient to unzip the sister chromatids of this arm by pulling of the centromeres (Guacci et al. 1994; Lavoie et al. 2004; Machín et al. 2005; Harrison et al. 2009). Taking into account this fact, together with the deficiencies in the spindle elongation and SPB positioning in *cdc14*-*ts* mutants, it seems conceivable that the failure to segregate the rDNA is just a matter of insufficient pulling forces by the spindle at this locus. This simple explanation could also be valid for other distal regions and telomeres in long chromosome arms. In agreement with this hypothesis, the cXIIr sisters appear partly resolved (i.e., two separated signals under the resolution of fluorescence microscopy), from the centromere to around one-third within the centromere-proximal rDNA, with the other two-thirds of the rDNA and the rest of the chromosome arm up to the telomere unresolved (Machín et al. 2005). The centromeric regions of both sisters appear fully segregated and close to the corresponding SPB, therefore, the outcome of this partial resolution is the formation of an interesting type of Anaphase Bridge (AB) (Fig. 1). This AB (cXIIr-AB hereafter) comprises two partly resolved sister chromatids as opposed to the classical AB of the McClintock’s breakage-fusion-bridge cycle envisioned for dicentric chromosomes (McClintock 1939). According to this hypothesis, Cdc14 would drive cXIIr segregation by loading condensin onto the rDNA to hypercondense this locus (more than the calculated average compaction) and, of course, by the final polar positioning of the SPBs and enlargement of the spindle. Experimental support for this hypothesis comes from different angles. First, we demonstrated that restoration of Cdc14 activity was enough to resolve and segregate the rDNA in many cells, even after previously depolymerizing the spindle (Machín et al. 2005). Second, rDNA is in a transient hypercondensed state in the window between FEAR and MEN (Guacci et al. 1994; Varela et al. 2009). Third, telomeres of other chromosomes with short arms are fully segregated in the *cdc14*-*1* (Quevedo et al. 2012). Fourth, recent findings with chimeric constructions of giant chromosome arms have shown a correlation between arm length and the time of sister chromatid resolution, although the role of Cdc14 was not tested directly (Titos et al. 2014).

Nevertheless, the simple solution introduced above for the occurrence of this cXIIr-AB seems to be actually more complicated. First, sixfold shortening of the rDNA array, which reduces the cXIIr size below the length limitation mentioned above, only partly improved cXIIr segregation (Machín et al. 2006; Tomson et al. 2006). Second, there are examples of telomeres other than cXIIr that are unresolved in the *cdc14*-*ts* blocks, including telomeres in relatively medium-sized arms (D’Amours et al. 2004; Clemente-Blanco et al. 2011). Third, incomplete replication within the rDNA has also been reported after Cdc14 depletion (Dulev et al. 2009). Fourth, condensin mutants also fail in resolving the rDNA (Freeman et al. 2000), and expression of an exogenous Topoisomerase II can overcome this defect (D’Ambrosio et al. 2008). Both, catenations and unfinished replication can physically prevent resolution of sister chromatids, and they have been shown to lead to ABs (Holm et al. 1985; Torres-Rosell et al. 2007; Germann et al. 2014). In addition, other DNA-mediated post-replication linkages such as DNA repair Joint Molecules (JMs) might preclude sister chromatid resolution. Of note, the rDNA is a hotspot for JMs due to the presence of naturally occurring replication fork blocks (RFB) and the rDNA high transcription rates. In putative agreement with a role of JMs in preventing Cdc14-mediated rDNA resolution, we showed some years ago that deletion of *FOB1*, the gene that encodes for the RFB determinant, worsened rDNA segregation in *cdc14*-*1* (Machín et al. 2006). The absence of Fob1 makes replication and transcription collide, which would in theory increase the frequency of both catenations and JMs (reviewed in Bermejo et al. 2012; Aguilera and Gaillard 2014), although the latter counter-intuitively seems to depend on the presence of Fob1 as well (Kobayashi and Horiuchi 1996). Besides this, we and others have recently shown that Cdc14 targets one important JM resolution endonuclease, Yen1, to the nucleus in a FEAR-dependent manner (Eissler et al. 2014; Blanco et al. 2014; García-Luis et al. 2014). Likewise, we have also demonstrated recently that unresolved JMs at the rDNA form an AB similar to that seen in *cdc14*-*1* (García-Luis and Machín 2014). Despite all these indications, it seems unlikely that JMs are behind the lack of rDNA resolution in the *cdc14*-*1* block; we only observed JM-related cXIIr ABs in *yen1*Δ when another JM-resolving endonuclease, Mus81-Mms4, is absent. Moreover, the cXIIr-AB levels in *yen1*Δ *mms4*Δ are comparable to *cdc14*-*1* only when cells concomitantly suffer from exogenous replication stress (García-Luis and Machín 2014).

A major breakthrough in the understanding on the role of Cdc14 in the resolution and segregation of cXIIr was the discovery of the in vitro and in vivo capability of Cdc14 to inhibit RNA polymerase I (Machín et al. 2006; Tomson et al. 2006; Clemente-Blanco et al. 2009), and that this step was needed to load condensin onto the rDNA (Clemente-Blanco et al. 2009). Once loaded onto the rDNA, condensin will compact the array and, together with Topoisomerase II, remove catenations and/or finish replication (Guacci et al. 1994; Freeman et al. 2000; D’Amours et al. 2004; Sullivan et al. 2004; Wang et al. 2004; Machín et al. 2006; Dulev et al. 2009).

Finally, one of the most obvious causes that might explain sister chromatid resolution failure, i.e., incomplete removal of the cohesin complex, was the first to be ruled out (D’Amours et al. 2004; Sullivan et al. 2004). Nevertheless, a few years ago it was shown that some cohesins escape separase action on yeast chromosomes arms and that condensin helps removing them (Renshaw et al. 2010). Thus, it is still possible that cohesin keeps the cXIIr-AB in the *cdc14*-*1* block after all.

## Without Cdc14 the right arm of chromosome XII forms an anaphase bridge: the consequences

An interesting result we reported in 2006 was that lowering the temperature back to permissive conditions (25 °C) once the cells are blocked at the *cdc14*-*1* arrest (37 °C) provided enough Cdc14 activity to repositioning the SPBs, elongating the spindle, and allowing exit from mitosis (Machín et al. 2006). However, the re-entry into the cell cycle occurs with around 50 % of cells failing to properly resolve the cXIIr-AB. This failure is not because the stretched cXIIr-AB breaks apart. We have not found evidence for such breaks at the time of the *cdc14*-*1* block by pulse-field gel electrophoresis (PFGE) (Quevedo et al. 2012). Furthermore, breakage of the bridge in anaphase, while CDK is still high, was expected to elicit a DNA damage response that we did not see (Quevedo et al. 2012). We hypothesize instead that the sister chromatid linkages that maintain the cXIIr-AB become irresolvable in late anaphase because (1) they change their physical structure, (2) concentrate on specific regions until they sterically suppress the action Cdc14, condensin and/or Top2, or (3) migrate towards new locations where these resolving factors do not normally operate. In support of this latter scenario, we observed that the absence of Fob1, which binds exclusively to the rDNA, led to failed resolution of the cXIIr telomere after the *cdc14*-*1* release, but the rDNA still got fully resolved in 50 % of the cells (Machín et al. 2006). In other words, the extra linkages that arose at the rDNA by the absence of Fob1 migrated towards the telomere upon Cdc14 activation.

Irrespective of the causes behind the maintenance of the cXIIr-AB after the *cdc14*-*1* release, we noticed that this situation could be exploited to address the consequences of this novel cXIIr-AB for the immediate progeny. Thus, we reported in 2012 that the cXIIr-AB is severed as cytokinesis seemed to proceed to completion, giving rise to two daughter cells with different genome contents (Quevedo et al. 2012). Of note, the severing of the bridge in haploids produces two one-ended Double-Strand Breaks (DSBs), one in each daughter cell (Fig. 1). Interestingly, both daughters trigger the Rad9-dependent DNA damage checkpoint in the next S-phase, not earlier on in G_1_, and try to repair the one-ended DSBs through the homologous recombination (HR) pathway. This happens in both daughters despite one of them is to become unviable as it has lost essential genetic information (Fig. 1). The other daughter cell, which carries an intact copy of cXII, often survives. It is still unclear if genetic rearrangements arise during its recovery, but preliminary data on a few instances suggest that the haploid surviving cells get rid of the acentric broken piece of the second chromatid rather than either translocating it to another chromosome or becoming disomic for cXII by copying the rest of the missing sister through break-induced replication (BIR) (Fig. 1).

**Fig. 2:**
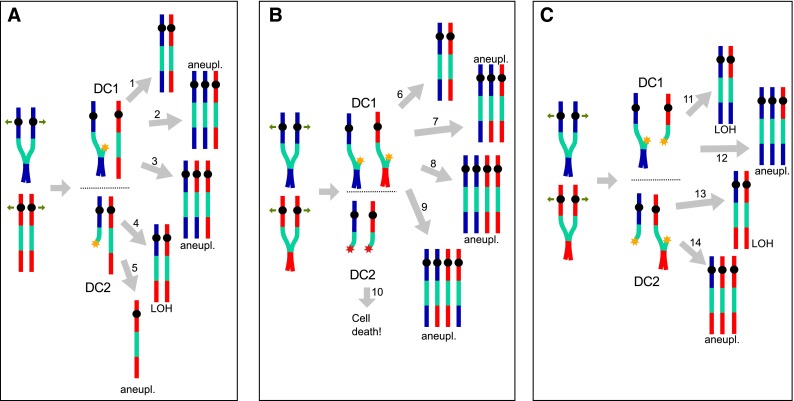
Putative outcomes in diploid cells of the breakage and repair of the chromosome XII right-arm anaphase bridge (cXIIr-AB). Homologs for chromosome XII are colored in *red* and *blue*. The rDNA is in *green*, *black circles* are centromeres and *yellow *and *red stars* are one-ended DSBs once the cXIIr-AB is severed by cytokinesis. DC1 and DC2 stand for daughter cell one and two (we prefer to use this terminology since the broken pieces of cXIIr-AB seem to segregate randomly between the mother and the daughter cells). *Numbered gray arrows*, different mechanisms whereby daughter cells deal with the broken cXII. **a** Just one cXII homolog is affected by the cXIIr-AB. DC1 receives the intact sister chromatid for that homolog and the acentric piece of the second sister. DC2 gets the remaining centromere-containing fragment. DC1 might deal with the DSB by getting rid of the acentric bit (*1*). This would be the ideal solution as DC1 will stay unchanged; i.e., disomic and heterozygous. DC1 might also repair the one-ended DSB through BIR and become trisomic (*2* or *3*). If BIR takes place with the intact sister (sc-BIR), DC1 would be trisomic without LOH (*2*). If BIR uses the homolog as template (hc-BIR) a terminal LOH would be present in the extra cXII (*3*). DC2 has two choices: An hc-BIR that would result in a terminal LOH event with retention of the euploid chromosome number (*4*) or, else, the loss of the broken sister leading to a cXII monosomy (*5*). **b** Both homologs form a cXIIr-AB. Upon the bridge breakage, DC1 gets both intact sister chromatids and both acentric fragments. DC2 just gets the two centric fragments and thus loses essential genetic information to proliferate. DC1 can progress further if it manages to get rid of both acentric broken fragments (*6*). Again, this situation would be ideal since it assures an intact euploid genome. Alternatively, DC1 can get rid of just one acentric fragment and use the other one for BIR, leading to a trisomy (*7*). The trisomy can come with an associated LOH (*7*) or not (like *arrow 2* in *panel* **a**) depending on whether hc-BIR or sc-BIR takes place. If there is BIR involving both acentric fragments, tetrasomy will be observed. Multiple rearrangements are possible in terms of associated terminal LOHs depending on the hc-BIR/sc-BIR combinations (*8*, *9* and others not depicted). **c** Both homologs form a cXIIr-AB. Upon the bridge breakage, each DC gets one intact sister chromatid, the entangled acentric fragment of the other sister, and the centromere-containing fragment of the broken sister from the other homolog. Since DSBs are expected at the repetitive rDNA, Single Strand Annealing (SSA) between the acentric and the centromere-containing fragments seems the straightforward solution. This would lead to a terminal LOH event with retention of the euploid chromosome number (*11*, *13*). A similar outcome will take place if both DSBs are repaired through the canonical HR pathway. BIR-based solutions for DC1 and DC2 would lead to trisomies with or without LOH as described above (*12*, *14*)

The limitations of haploids to assess the putative genomic rearrangements that followed the severing of the cXIIr-AB prompted us to recently study this phenomenon in diploids. Importantly, diploid cells also form the cXIIr-AB, although the failure to timely resolve the bridge after the *cdc14*-*1* release is even worse than in haploids (Quevedo et al. 2015). Also striking is the fact that the viability is even lower despite the existence of additional templates for repair (the chromatids from the other homolog) (Fig. 2). Single Nucleotide Polymorphism (SNP) microarray studies in a highly heterozygous hybrid *cdc14*-*1*/*cdc14*-*1* strain showed that cXII is frequently reorganized and Loss Of Heterozygosity (LOH) events, trisomies and monosomies were often found in surviving colonies. Most of these genetic rearrangements fit well within the predictions of models to deal with the broken cXIIr-AB (Fig. 2). It was surprising, though, that chromosomes other than cXII were often rearranged since anaphase bridges involving other chromosomes were less expected. There are many putative causes for these extra rearrangements and we discussed them in detail in the recent manuscript (Quevedo et al. 2015). In addition to the LOH events on chromosomes other than XII, we observed ploidy alterations. Interestingly, trisomies for small chromosomes were among the most frequent genetic changes.

## Concluding remarks and future perspectives

The study of Cdc14 has provided important insights into the control of the cell cycle and the counterbalance of CDK activities. Of the many phenotypes observed in cells transiently deficient for Cdc14, the formation of an anaphase bridge that comprises partly resolved sister chromatids has unexpectedly provided a model to study the consequences for the progeny of this aberrant situation (Machín et al. 2005, 2006; Quevedo et al. 2012, 2015). Anaphase bridges of this nature have long been suspected, and there seems to be a connection with cancer and other human diseases. It remains to be determined if Cdc14 homologs in humans (up to three) play roles in preventing their occurrence. In this respect, hCdc14A deregulation has been shown to cause chromosome segregation problems, although probably through its role in centrosome dynamics (Kaiser et al. 2002; Mailand et al. 2002). Also, it should be tested if the main CDK-counteracting phosphatases in humans, PP1 and PP2A, are the ones that play some role instead as it suggests the PP2A regulation on centromeric cohesion (Tang et al. 2006). In yeast, this anaphase bridge model should be expanded to other situations that do not depend on Cdc14. The new tools to make chimeric giant chromosomes could be a good approach, although strategies to make such chromosomes conditionally need to be developed. As for yeast Cdc14, the new results in diploid cells raised new questions about the events triggered by Cdc14 in anaphase that prevent the accumulation of trisomies (and other genetic instabilities) in the progeny. We foresee exciting years ahead in the understanding of how anaphase bridges reshape the genome.
